# Comparison of Homeostasis Model Assessment-Insulin Resistance (HOMA-IR) and the Level of Cortisol Between Preterm and Term Newborns

**DOI:** 10.7759/cureus.32623

**Published:** 2022-12-17

**Authors:** Ravivarma Ramalingam, Sunanda Jha, Upendra Prasad Sahu, Bhardwaj Narayan Chaudhary, Surabhi Baxla, Pawan Kumar, Surekha R, Akanksha Kumari

**Affiliations:** 1 Department of Pediatrics, Rajendra Institute of Medical Sciences, Ranchi, IND

**Keywords:** fetal hyperinsulinemia, homeostatic model assessment of insulin resistance (homa-ir), term newborns, thrifty hypothesis, glucose, cortisol, insulin, insulin resistance

## Abstract

Introduction

According to the thrifty (Barker’s) phenotype hypothesis, poor nutrition in fetal and early infancy plays a role in the development and function of the beta cells of the islets of Langerhans, which leads to the development of type 2 diabetes mellitus. Insulin resistance is due to decreased suppressive effect of insulin on hepatic glucose production. Thus, elevated insulin levels during perinatal life may predispose the infant to the development of diabetes mellitus in future life. Intrauterine undernutrition plays an important role in causing adult insulin resistance and diabetes but the exact cause is still unknown. Preterm infants born small for gestational age (SGA) show lower adrenocortical response to stimulation due to an immature hypothalamic-pituitary axis.

Methods

The cross-sectional study conducted at Rajendra Institute of Medical Sciences, Ranchi from June 2020 to November 2021 included 216 newborns enrolled as per the inclusion and exclusion criteria. Maternal and neonatal details were collected at birth and recorded. Cord blood samples for measurement of serum insulin, glucose, and cortisol were collected from 84 preterm and 132 term neonates. Using this information, homeostasis model assessment-insulin resistance (HOMA-IR) was calculated using a mathematical formula. Insulin resistance was defined as HOMA-IR > 2.5. Based on birth weight and gestational age, they were further categorized into SGA, appropriate for gestational age (AGA), and large for gestational age (LGA). The parametric data were presented as means ± standard deviation (SD), and nonparametric data as medians (first quartile and third quartile). The Student’s (independent samples) t-test and Mann-Whitney U test were used to compare mean differences between the two groups for parametric and nonparametric data, respectively. The Spearman correlation coefficient was used to determine the significant association between variables.

Results

Umbilical cord plasma glucose and serum insulin were high in preterm in comparison to term newborns. Serum cortisol levels were high in term than in preterm newborns. HOMA-IR showed a very strong positive correlation with serum insulin and a moderate positive correlation with serum glucose. HOMA-IR showed a strong negative correlation with gestational age and a moderate negative correlation with birth weight. Insulin resistance was seen in 34 preterm newborns and two term newborns. Insulin resistance was seen in 29.8% (n = 25) of SGA preterm babies, 7.1% (n = 6) of AGA preterm babies, and 1.5% (n = 2) of AGA term newborns. A total of 55.6% of newborns were below normal weight (48.1% had low birth weight, 4.6% had very low birth weight, and 2.8% had extremely low birth weight).

Conclusion

Our study suggests that preterm newborns are more insulin resistant at birth than term newborns. SGA preterm babies are having a higher incidence of insulin resistance compared to AGA preterm babies. It is clear that high insulin level is needed to overcome high insulin resistance in the very early gestational period. Serum cortisol increases as gestational age and birth weight increase. Thus, serum cortisol helps in the maturation of the fetus and neonatal adaptation at birth.

## Introduction

The fetus receives glucose almost entirely from the mother through placental transfer under normal conditions. It becomes completely independent for glucose homeostasis after the umbilical cord is severed. The glucose needs of the newborn are three to four times higher than that of adults on a per body weight basis. This high requirement of glucose during the newborn period is mainly due to higher rates of cerebral glucose utilization. This is controlled smoothly by coordinated changes in concentrations of insulin and the counter-regulatory hormones, mainly growth hormone, cortisol, glucagon, and epinephrine [1].

Because of the immaturity of the glucose homeostatic pathways in the neonate, there are transient disturbances in the glucose homeostasis soon after birth. The neonates are more frequently faced with hypoglycemia, which is more commonly seen in preterm babies and neonates with intrauterine growth restriction, as glycogen reserves are low [2]. Preterm infants have limited glycogen stores [3]. Due to limited metabolic fuel availability and/or uteroplacental insufficiency, growth-restricted fetuses use glucose for growth and not for glycogen synthesis [4]. Glucokinase is decreased in preterm infants due to relative defects in insulin activity and or sensitivity. Insulin-sensitive tissues like adipose tissue, skeletal muscle, and cardiac muscle are less abundant in preterm than in term neonates, thereby leading to hyperglycemia by poor uptake of glucose [5].

According to the thrifty (Barker’s) phenotype hypothesis [6], poor nutrition in fetal and early infancy plays a role in the development and function of the beta cells of the islets of Langerhans, which leads to the development of type 2 diabetes mellitus. Insulin resistance is due to decreased suppressive effect of insulin on hepatic glucose production. Thus, elevated insulin levels during perinatal life may predispose the infant to the development of diabetes mellitus in future life. Intrauterine undernutrition plays an important role in causing adult insulin resistance and diabetes but the exact cause is still unknown. The objective of our study was to compare the serum cortisol, serum glucose, serum insulin, and homeostasis model assessment-insulin resistance (HOMA-IR) index between preterm and term newborns at birth.

## Materials and methods

The study was approved by the Rajendra Institute of Medical Sciences (RIMS), Institutional Review Board and Ethics Committee (IEC-194). Informed written consent was obtained from the parents of all the participants at recruitment into the study. The sample size was calculated using the formula for comparing two independent means. The minimum expected difference in the level of HOMA-IR between preterm and term newborns was 0.5 with an SD of 1.25 at a 5% level of significance and 90% power. The recruitment and collection of data at birth were done in the labor room of RIMS, Ranchi. Recruitment for the study commenced in June 2020. The major recruitment occurred between November 2020 and November 2021. Due to the COVID-19 pandemic, the study got interrupted due to the lockdown implementation.

The study included preterm neonates born between 24 weeks and 36^6/7^ weeks of gestation and term neonates born between 37 weeks and ≤42 weeks of gestational age. Those newborns born through normal vaginal delivery at RIMS, Ranchi with APGAR (appearance, pulse, grimace, activity, and respiration) at one minute ≥ 7 underwent estimation of cord blood for glucose, insulin, and cortisol. Table 1 shows the inclusion and exclusion criteria.

**Table 1:** Inclusion and exclusion criteria APGAR: appearance, pulse, grimace, activity, and respiration; PCOD: polycystic ovarian disease.

S. No.	Inclusion criteria	Exclusion criteria
1	Newborns born through normal vaginal delivery at Rajendra Institute of Medical Sciences, Ranchi	Newborns born through cesarean section, assisted vaginal delivery including instrumental delivery, vacuum delivery, meconium aspiration syndrome
2	Term newborns between 37 and 42 weeks	Mothers with obstetric complications like gestational diabetes, hypertension, kidney disease, PCOD, prolonged rupture of membranes, abruptio placenta, smoking, alcohol, mothers with a need for dextrose solution, etc.
3	Preterm newborns between 24 and 36^6/7 ^weeks	Newborns with an abnormal presentation, lie, footling, major congenital anomalies, chromosomal anomalies, genetic syndromes, etc.
4	APGAR at 1 minute ≥ 7	Incomplete/missing maternal details

Neonates born at RIMS hospital and who met the inclusion criteria were grouped into two categories: preterm with gestational age between 24 and 36^6/7^^ ^weeks and term with gestational age between 37 and 42 weeks. They were further subcategorized as small for gestational age (SGA), appropriate for gestational age (AGA), and large for gestational age (LGA).

Study procedure

Venous cord blood was collected after delivery but prior to the expulsion of the placenta and 3 ml was drawn into plain and fluoride vacutainers. Plasma glucose was analyzed within four hours of collection by glucose oxidase peroxidase method in an autoanalyzer whereas serum cortisol and serum insulin were analyzed by enzyme-linked immunosorbent assay with a sample from the plain vacutainer. The normal cord blood glucose, cortisol, and insulin were 45-96 mg/dl, 1-24 mcg/dl, and 3-20 mIU/ml, respectively [7,8]. Based on expanded new Ballard scoring, the gestational age was used to categorize preterm newborns and term newborns. It was further subcategorized as SGA, AGA, and LGA. Insulin resistance was defined as HOMA-IR > 2.5 [9,10]. A mathematical formula was used to calculate HOMA-IR [11]: HOMA-IR = serum glucose concentration (mg/dl) x serum insulin concentration (mIU/ml)/405.

Data entry and analysis

All data were entered using Microsoft Excel (Microsoft Corporation, Redmond, WA) and analyzed using the IBM SPSS Statistics version 20 (IBM Corp., Armonk, NY). The parametric data were presented as means ± standard deviation (SD) and nonparametric data as medians (first quartile and third quartile). The Student’s (independent samples) t-test and Mann-Whitney U test were used to compare mean differences between the two groups for parametric and nonparametric data, respectively. The Spearman correlation coefficient was used to determine the significant association between variables. All statistical analyses were carried out at a 5% level of significance and p < 0.05 was considered significant.

## Results

In this cross-sectional study of 216 newborns, there were 84 (38.9%) preterm and 132 (61.1%) term neonates. Newborn and maternal demographics are presented in Tables 2, 3.

**Table 2:** Newborn demographics SGA: small for gestational age; AGA: appropriate for gestational age; LGA: large for gestational age.

Newborn demographics	
Total newborns (n = 216)	N (%)
Male	125 (57.9)
Female	91 (42.1)
Birth weight (n = 216)	
Extreme low birth weight (<1000 g)	6 (2.8)
Very low birth weight (1000-1499 g)	10 (4.6)
Low birth weight( 1500-2499 g)	104 (48.1)
Normal birth weight (2500-3999 g)	96 (44.4)
Gestational age (n = 216)	
Term (37-42 weeks)	132 (61.1)
Preterm (28-36^6/7 ^weeks)	84 (38.9)
Early preterm (28-31^6/7^ weeks)	24 (11)
Moderate preterm (32-33^6/7^ weeks)	24 (11)
Late preterm (34-36^6/7 ^weeks)	36 (16.9)
Preterm (n = 84)	
SGA	48 (57)
AGA	35 (42)
LGA	1 (1)
Term (n = 132)	
SGA	73 (55)
AGA	59 (45)

**Table 3:** Maternal demographics Q1: first quartile; Q3: third quartile.

Maternal demographics (n = 216)	
Mother’s age in years, median (Q1, Q3)	22 (20, 25)
Father’s age in years, median (Q1, Q3)	24 (23, 27)
Gravida (n = 216)	
Primigravida, n (%)	128 (59.3)
Second gravida, n (%)	55 (25.5)
Third gravida, n (%)	21 (9.7)
Fourth gravida, n (%)	11 (5.1)
Fifth gravida and above, n (%)	1 (0.5)
Parity (n = 216)	
Primipara, n (%)	143 (66.2)
Second parity, n (%)	51 (23.6)
Third parity and above, n (%)	22 (10.2)
Tetanus toxoid injection not taken even single dose, n (%)	32 (14.8)
Iron and folic acid tablets not taken during pregnancy, n (%)	49 (22.7)

Cord blood glucose in preterm and term newborns

The mean serum glucose in preterm newborns was higher than the term newborns, which was statistically significant using the independent Student's t-test, as shown in Table 4.

**Table 4:** Mean serum glucose in preterm and term babies Serum glucose is expressed in mean (SD).

Parameters	Preterm	Term	P-value
Serum glucose, mg/dl	109.45 (19.6)	98.47 (21.91)	<0.001

Cord blood insulin and cortisol in preterm and term newborns

Using the Mann-Whitney U test, the median of serum cortisol was significantly higher in term newborns while the median of serum insulin was significantly higher in preterm newborns, as shown in Table 5.

**Table 5:** Comparison of serum cortisol and serum insulin in preterm and term newborns Serum cortisol and insulin are expressed in median (Q1, Q3).

Parameters	Preterm	Term	P-value
Serum cortisol (µg/dl)	10.6 (7.2, 13.2)	12 (8-18)	<0.01
Serum insulin (mIU/ml)	8.9 (6.4, 12)	2.8 (1.8-3.8)	<0.05

HOMA-IR in preterm and term newborns

HOMA-IR was calculated using the mathematical formula. HOMA-IR > 2.5 was considered the cut-off for insulin resistance [9,10]. Insulin resistance was higher in preterm than term newborns using the Mann-Whitney U test, as shown in Table 6. Among the preterm newborns (n = 84), insulin resistance was seen in 31 newborns. Insulin resistance was found to be more common in SGA preterm babies (n = 25, 29.8%) than in AGA preterm babies (n = 6, 7.1%). Among the term newborns (n = 132), insulin resistance was seen in two (1.5%) AGA term newborns.

**Table 6:** Comparison of HOMA-IR between preterm and term newborns HOMA-IR is expressed in median (Q1, Q3). HOMA-IR: homeostasis model assessment-insulin resistance.

Parameter	Preterm	Term	P-value
HOMA-IR	2.4 (1.6-3.2)	0.7 (0.4-1)	<0.01

Correlation of serum insulin, serum cortisol, serum glucose, gestational age, birth weight, and HOMA-IR

Spearman rank order correlation was used to find out the association between two non-parametric variables, as shown in Table 7.

**Table 7:** Correlation of serum insulin, serum cortisol, serum glucose, gestational age, birth weight, and HOMA-IR HOMA-IR: homeostasis model assessment-insulin resistance.

	Gestational age	Birth weight	Serum insulin	Serum cortisol	Serum glucose	HOMA-IR
Gestational age	1	0.719	-0.652	0.124	-0.236	-0.654
Birth weight	0.719	1	-0.533	0.105	-0.313	-0.563
Serum insulin	-0.652	-0.533	1	-0.031	0.297	0.974
Serum cortisol	0.124	0.105	-0.031	1	-0.122	-0.053
Serum glucose	-0.236	-0.313	0.297	-0.122	1	0.479
HOMA-IR	-0.654	-0.563	0.974	-0.053	0.479	1

We found that gestational age showed a strong positive correlation with birth weight, a strong negative correlation with serum insulin levels and HOMA-IR, and a weak negative correlation with serum glucose. Birth weight showed a strong positive correlation with gestational age, a moderate negative correlation with serum insulin and HOMA-IR, and a weak negative correlation with serum glucose. Serum insulin showed a very strong positive correlation with HOMA-IR and a weak positive correlation with serum glucose. Serum insulin also showed a strong negative correlation with gestational age and a moderate negative correlation with birth weight. Serum cortisol was found to have a very weak positive correlation with birth weight and gestational age and a very weak negative correlation with serum insulin, serum glucose, and HOMA-IR. Serum glucose was found to have a moderate positive correlation with HOMA-IR and a weak positive correlation with serum insulin. Serum glucose showed a weak negative correlation with gestational age and birth weight. HOMA-IR showed a very strong positive correlation with serum insulin and a moderate positive correlation with serum glucose. HOMA-IR showed a strong negative correlation with gestational age and a moderate negative correlation with birth weight.

## Discussion

In this study, we compared the levels of cord blood glucose, insulin, and cortisol in term and preterm newborns. We also compared the insulin resistance in term and preterm newborns using the HOMA-IR formula. The levels of serum glucose, serum cortisol, and serum insulin were 102.7 mg/dl (58-173 mg/dl), 11.3 μg/dl (interquartile range (IQR): 7.2-18 μg/dl), and 5.9 mIU/ml (IQR: 1.8-12 mIU/ml), respectively. In preterm newborns, the mean serum glucose (109.5 ± 19.6 mg/dl) was higher than the term newborns (98.5 ± 21.9 mg/dl), which is higher than the expected normal range [7,8]. Few other studies reported plasma glucose and insulin levels to be higher in preterm than term newborns [12-14]. In such hyperglycemic newborns, pancreatic β cells are sensitive to changes in blood glucose concentration. But in response to hyperglycemia, these pancreatic β cells increase the secretion of the non-processed proinsulin [15]. We already know that proinsulin is 10-fold less active than mature insulin and does not control plasma glucose levels. Hyperglycemia is mainly related to defective islet β-cell processing of proinsulin. More evidence in the past had shown that preterm newborns are partially resistant to insulin action due to higher proinsulin than insulin. Studies by Farrag et al. [5] concluded that insulin unresponsiveness in hyperglycemic preterm newborns may be related to a post-receptor defect. During hyperglycemia, persistent gluconeogenesis may not be the cause of continuous glucose production. Glucokinase, an insulin-dependent enzyme, is needed for the utilization of glucose. But this enzyme is decreased in the liver of preterm newborns [16]. This might lead to excessive release of glucose into the circulation because hepatocytes are unable to metabolize glucose efficiently. Also, these preterm newborns have less insulin-dependent tissues like adipose tissue and skeletal and cardiac muscles. This might lead to diminished peripheral glucose uptake leading to hyperglycemia.

Various studies by Gesteiro et al. [17], Kırımi et al. [7], and Sano et al. [18] showed that cortisol levels were 4.4-10.4 μg/dl, 5.73-21.5 μg/dl, and 70-313 ng/ml, respectively, in full-term newborns. A study by Ahmad et al. [19] showed that serum cortisol in preterm and term newborns was 8.9 ± 4.66 μg/dl (4.4-24 μg/dl) and 11.88 ± 5.78 μg/dl (3-26 μg/dl), respectively. Our serum cortisol findings in preterm and term newborns corroborate with other published findings. This shows that cortisol helps in the maturation of the fetus and neonatal adaptation at birth [20]. Fetal cortisol levels remain low until 30 weeks and rise near late term [7]. Even though in our study, the median for cortisol showed a statistical difference between preterm and term newborns, we did not find any significant statistical correlation between birth weight and gestational age. Similar findings were seen in a study done by Bagnoli et al. [21], which showed no correlation between gestational age and cortisol. Perinatal stress during labor/delivery may be attributed to increased cortisol secretion in preterm but to a lesser extent in term newborns.

In different studies carried out by Kırımi et al. [7], Sahasrabuddhe et al. [22], and Ahmad et al. [19], serum insulin was 6.01 ± 3.67 mIU/ml (0.5-18 mIU/ml), 6.75 ± 2.96 mIU/ml, and 8.3 ± 2.1 mIU/ml, respectively, in term newborns. Ahmad et al. [19] reported serum insulin to be 13.7 ± 4.7 in preterm babies. With respect to preterm babies, our findings concurred with the findings of Bagnoli et al. [23], in which insulin levels were higher in very preterm than in late preterm (8.61 ± 2.48 vs. 3.98 ± 0.94 mU/L) and full-term infants (8.61 ± 2.48 vs. 4.56 ± 1.2 mU/L). Our study results did not match the results from Akinola et al.'s [24] study, in which serum insulin in term newborns was estimated to be 8.68 ± 3.63 mIU/ml. They also noted that insulin levels in LGA, AGA, and SGA were 9.47 ± 4.77 mIU/ml, 8.44 ± 2.03 mIU/ml, and 7.98 ± 3.08 mIU/ml, respectively. We also found that serum insulin is significantly negatively correlated with gestational age and birth weight. It is clear that insulin in umbilical cord blood is of fetal origin since maternal insulin does not cross the placenta [25]. It is clear that high insulin level is needed to overcome high insulin resistance in a very early gestational period [23]. This resistance improves over time with an increase in receptor number and maturity as well as peripheral sensitivity to insulin [26]. Lastly, down-regulation of GLUT4 expression in very preterm infants can lead to insulin resistance. HOMA-IR was used to calculate insulin resistance. We found that 31/84 preterm newborns had insulin resistance (HOMA-IR > 2.5). HOMA-IR was found to be more in SGA preterm babies (25/84, 29.8%) than in AGA preterm babies (6/84, 7.1%). Two (1.5%) of 132 AGA term newborns were found to have insulin resistance.

There are very few studies that report HOMA-IR in cord blood [19]. We report that HOMA-IR is negatively correlated with gestational age and birth weight. Similar results were observed by Sahasrabuddhe et al. [22] and Wang et al. [27]. Serum insulin was found to have a significant positive correlation with HOMA-IR. This also concurred with the results of Ahmad et al. [19]. Even though we found that cortisol was positively correlated with gestational age and birth weight, they were not statistically significant. We also found that HOMA-IR was negatively correlated with cortisol. However, there was no significant statistical correlation between cortisol and HOMA-IR. In our study, we found out that 57% were SGA preterm and 55% were SGA term newborns. Mericq [28] reported that IUGR was associated with increased insulin levels rather than low birth weight itself.

Limitations

Our study comprised mainly SGA and AGA babies, which eluded us from studying insulin resistance and other parameters related to LGA babies. Since most of the newborns were SGA in both preterm and term newborn groups, statistical significance in serum cortisol between the two groups could not be ascertained. Long-term follow-up of these newborns could not be done, which would have helped us to know if these newborns developed insulin resistance or metabolic syndrome in childhood.

Strengths

Our study involved a large sample size for the estimation of HOMA-IR and other parameters in cord blood. Also, this was the first study comparing cord blood HOMA-IR and cortisol in newborns of various gestations in the eastern part of India. An additional contribution of our study was the reference values of cord insulin levels and HOMA-IR in the Indian population for future studies.

## Conclusions

Our study suggests that preterm newborns are more insulin resistant at birth than term newborns. SGA preterm babies are having a higher incidence of insulin resistance compared to AGA preterm babies. It is clear that high insulin level is needed to overcome high insulin resistance in the very early gestational period. Serum cortisol increases as gestational age and birth weight increase. Thus, serum cortisol helps in the maturation of the fetus and neonatal adaptation at birth. Newborns with insulin resistance in this study need to be followed up at regular intervals for the development of diabetes mellitus and metabolic syndrome in the future. Strengthening antenatal care can prevent the incidence of low birth weight babies and prematurity.

## References

[1] Hay WW Jr (2006). Placental-fetal glucose exchange and fetal glucose metabolism. Trans Am Clin Climatol Assoc.

[2] Ward Platt M, Deshpande S (2005). Metabolic adaptation at birth. Semin Fetal Neonatal Med.

[3] Sharma A, Davis A, Shekhawat PS (2017). Hypoglycemia in the preterm neonate: etiopathogenesis, diagnosis, management and long-term outcomes. Transl Pediatr.

[4] Rao PN, Shashidhar A, Ashok C (2013). In utero fuel homeostasis: lessons for a clinician. Indian J Endocrinol Metab.

[5] Farrag HM, Nawrath LM, Healey JE, Dorcus EJ, Rapoza RE, Oh W, Cowett RM (1997). Persistent glucose production and greater peripheral sensitivity to insulin in the neonate vs. the adult. Am J Physiol.

[6] Neel JV (1962). Diabetes mellitus: a "thrifty" genotype rendered detrimental by "progress"?. Am J Hum Genet.

[7] Kırımi E, Cesur Y, Gül A (2000). Normal levels of insulin, growth hormone and cortisol levels in venous cord blood of healthy full-term infants: correlation with birthweight and placental weight. East J Med.

[8] Kliegman R, Stanton B, St Geme JW, Schor NF, Behrman RE, Nelson WE (2020). Nelson Textbook of Pediatrics. Nelson Textbook of Pediatrics. Edition 21 ed. Philadelphia PA: Elsevier.

[9] Simental-Mendía LE, Castañeda-Chacón A, Rodríguez-Morán M, Guerrero-Romero F (2012). Birth-weight, insulin levels, and HOMA-IR in newborns at term. BMC Pediatr.

[10] Singh Y, Garg MK, Tandon N, Marwaha RK (2013). A study of insulin resistance by HOMA-IR and its cut-off value to identify metabolic syndrome in urban Indian adolescents. J Clin Res Pediatr Endocrinol.

[11] Matthews DR, Hosker JP, Rudenski AS, Naylor BA, Treacher DF, Turner RC (1985). Homeostasis model assessment: insulin resistance and beta-cell function from fasting plasma glucose and insulin concentrations in man. Diabetologia.

[12] Hawdon JM, Aynsley-Green A, Alberti KG, Ward Platt MP (1993). The role of pancreatic insulin secretion in neonatal glucoregulation. I. Healthy term and preterm infants. Arch Dis Child.

[13] Tyrala EE, Chen X, Boden G (1994). Glucose metabolism in the infant weighing less than 1100 grams. J Pediatr.

[14] Mitanchez D (2007). Glucose regulation in preterm newborn infants. Horm Res.

[15] Hawdon JM, Hubbard M, Hales CN, Clark PM (1995). Use of specific immunoradiometric assay to determine preterm neonatal insulin-glucose relations. Arch Dis Child Fetal Neonatal Ed.

[16] Iynedjian PB, Jotterand D, Nouspikel T, Asfari M, Pilot PR (1989). Transcriptional induction of glucokinase gene by insulin in cultured liver cells and its repression by the glucagon-cAMP system. J Biol Chem.

[17] Gesteiro E, Bastida S, Sánchez-Muniz FJ (2009). Insulin resistance markers in term, normoweight neonates. The Mérida cohort. Eur J Pediatr.

[18] Sano Y, Doi T, Kikuchi S, Kawai K, Tanaka M (2015). Correlations between stress hormone levels in umbilical cord blood and duration of delivery. J Pak Med Assoc.

[19] Ahmad A, Srikantiah RM, Yadav C, Agarwal A, Manjrekar PA, Hegde A (2016). Cord blood levels of insulin, cortisol and HOMA2-IR in very preterm, late preterm and term newborns. J Clin Diagn Res.

[20] Hillman NH, Kallapur SG, Jobe AH (2012). Physiology of transition from intrauterine to extrauterine life. Clin Perinatol.

[21] Bagnoli F, Mori A, Fommei C, Coriolani G, Badii S, Tomasini B (2013). ACTH and cortisol cord plasma concentrations in preterm and term infants. J Perinatol.

[22] Sahasrabuddhe A, Pitale S, Raje D, Sagdeo MM (2013). Cord blood levels of insulin and glucose in full-term pregnancies. J Assoc Physicians India.

[23] Bagnoli F, Vodo F, Vodo S (2014). Glucagon and insulin cord blood levels in very preterm, late preterm and full-term infants. J Pediatr Endocrinol Metab.

[24] Akinola IJ, Oyenusi EE, Odusote OA, Oduwole AO, Njokanma FO (2018). Insulin resistance profile of apparently healthy term neonates in Lagos, Nigeria. J Clin Neonatol.

[25] Spellacy WN, Buhi WC (1976). Glucagon, insulin and glucose levels in maternal and umbilical cord plasma with studies of placental transfer. Obstet Gynecol.

[26] Mitanchez-Mokhtari D, Lahlou N, Kieffer F, Magny JF, Roger M, Voyer M (2004). Both relative insulin resistance and defective islet beta-cell processing of proinsulin are responsible for transient hyperglycemia in extremely preterm infants. Pediatrics.

[27] Wang Q, Wang XD, Liu X, Xiang Y, Liu HC, Zeng LK (2018). Effect of intrauterine growth retardation on insulin sensitivity and plasma adiponectin level in neonates. (Article in Chinese). Zhongguo Dang Dai Er Ke Za Zhi.

[28] Mericq V (2006). Prematurity and insulin sensitivity. Horm Res.

